# Substance use and associated factors among preparatory school students in Kolfe-Keranyo sub-city of Addis Ababa, Ethiopia

**DOI:** 10.1186/s12199-021-01032-1

**Published:** 2021-11-19

**Authors:** Leila Seid, Binyam Gintamo, Zelalem Negash Mekuria, Hussien Seid Hassen, Zemichael Gizaw

**Affiliations:** 1 Addis Ababa Medical and Business College, Addis Ababa, Ethiopia; 2 Department of Sociology and Social Work, College of Social Sciences and Humanity, Arsi University, Arsi, Ethiopia; 3 Department of Environmental and Occupational Health and Safety, Institute of Public Health, College of Medicine and Health Sciences, University of Gondar, Gondar, Ethiopia

**Keywords:** Substance use, Alcohol, Khat, Cigarette, Preparatory school students, Kolfe-Keraniyo sub-city, Addis Ababa, Ethiopia

## Abstract

**Background:**

Substance abuse is a worldwide problem that primarily affects adolescents, resulting in chronic health complications as well as psychosocial challenges and economic losses. However, the magnitude of the problem and the factors that contribute to it are not well studied in Ethiopia, particularly in the study area. As a result, this study was carried out to determine the prevalence and associated factors of substance use among preparatory school students in the Kolfe-Keraniyo sub-city of Addis Ababa, Ethiopia.

**Methodology:**

An institution-based cross-sectional study of 383 randomly selected preparatory school students in the Kolfe-Keraniyo sub-city was conducted. The data were gathered using a pretested self-administered structured questionnaire. Multivariable binary logistic regression analysis was employed to identify factors associated with substance use based on the adjusted odds ratio (AOR) and 95% confidence interval (CI) with *p* values less than 0.05.

**Result:**

This study revealed that the lifetime prevalence of substance use among preparatory students in Kolfe-Keraniyo sub-city, Addis Ababa, Ethiopia, was 26.5% (95% CI, 22.2, 30.7%). Specifically, 16% drunk alcohol, 9.6% smoked cigarette, and 9.4% chewed khat. The 16.3% were current users, of which 8.3% were drinkers, 6.4% were smokers, and 5.9% were khat chewers. Substance use was significantly associated with being male (AOR, 3.3; 95% CI, 1.284, 8.613), having alcohol drinking family member (AOR, 4.0; 95% CI, 1.704, 9.196), having khat chewing family member (AOR, 2.87; 95% CI, 1.161, 7.070), poor school substance use controlling rule (AOR, 6.64; 95% CI, 1.863, 23.687), availability of substance retailing shops in residential areas (AOR, 2.9; CI, 1.303, 6.606), strong relationship with parents (AOR, 0.005; 95% CI, 0.001, 0.026), and being member of school mini-media (AOR, 0.177; 95% CI, 0.048, 0.657).

**Conclusion:**

According to the findings of this study, one-quarter of the study participants were substance users. Alcohol, khat, and cigarettes were all commonly used substances. Gender, parent-child relationship, family member substance use history, school substance use controlling rules, school mini-media and pro-social involvement, and the availability of substance retailing shops were all strongly associated with substance use. Strengthening school rules on substance use, controlling substance retailing shops near schools and residential areas, and providing students with health education are all strategies for reducing substance use among students.

## Background

Substance abuse is a major public health concern that affects people of all ages and social classes, but it is especially dangerous among the young. Substance use disorder occurs when users use alcohol or other restorative substances, posing health risks to themselves or others. Despite differences in prevalence due to age, gender, and other factors, substance use affects all people of all ages, residents, and gender. The types of substances commonly used in one area by people of different ages may differ from one another. Substance abuse among adolescents is common and growing at an alarming rate in both rural and urban areas, according to research [1].

Substance use, according to the World Health Organization [2], refers to the use of various substances such as alcohol and other substances such as cigarettes, illegal drugs, prescription drugs, inhalants, and solvents. Hookah, khat, marijuana, cocaine, tobacco, cannabis, heroin, liquors like alcohol, and inhalants are also commonly used substances among adolescents. Hookahs are water pipes that are used to smoke flavored tobacco, such as apple, mint, cherry, chocolate, coconut, licorice, cappuccino, and watermelon [3]. Khat is a drug found in the leaves of a wild, East African shrub called *Catha edulis*. The plant contains a central nervous system stimulant called cathinone [4]. People frequently use these substances for a variety of reasons, including bringing about the desired mood change or mood highness, relaxing, staying alert while reading, avoiding fear, relieving stress, managing depression and loneliness, and improving interpersonal relationships among peers [5].

The commonly used substances among adolescents vary depending on geographic locations. In Ethiopia, the most used substances among students include locally produced substances (araki, tella, and tej) [5] and those affordable modern substances (beer, cigarette, draft, etc.). *Tella* is a popular Ethiopian traditional beverage, which is made from diverse ingredients or substrates such as barley, wheat, maize, millet, and sorghum [6–8]. *Areki* is a distilled, colorless, clear, traditional alcoholic beverage in which fermented products are prepared in almost the same way as tella except that the fermentation mass in this case is more concentrated [8, 9]. *Tej* is a home-processed and commercially available honey wine. Some *tej* producers also include different concoctions such as barks, roots of some plants, and herbal ingredients to improve flavor or potency of *tej* *[*7*,* 8*]*. While in the West and Asia, commonly used substances include alcohol, cigarette, cannabis, water pipes, and heroin [10]. Adolescents usually start substance use from cigarette smoking and alcohol consumption as social drinkers but later on develop into the consumption of dangerous substances with increased volume [11].

**Fig. 1:**
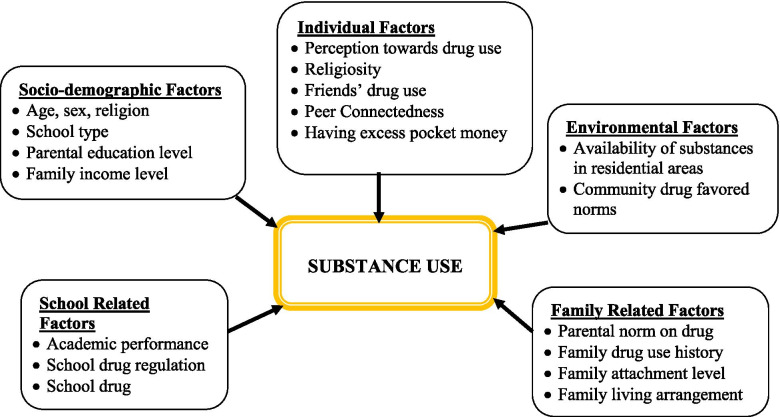
A conceptual framework shows factors associated with substance use

In developing countries, substance use was rare decades ago due to the positive influence of different indigenous cultural factors, but globalization and intercontinental population movement and associated cultural adaptation increased the prevalence [12]. Substance use among students is associated with individual factors, socio-demographic factors, family-related factors, school-related factors, and environmental factors [13, 14] (Fig. 1). However, the prevalence of substance use and contributing factors are not well studied in Ethiopia, particularly in the study area. This study was, therefore, conducted to assess the prevalence and associated factors of substance use among preparatory school students in Kolfe-Keraniyo sub-city, Addis Ababa, Ethiopia.

## Methods

### Study design and description of the study setting

An institutional-based cross-sectional study design was employed among preparatory school students in the Kolfe-Keraniyo sub-city. Kolfe-Keranio is one of the ten sub-cities of Addis Ababa and as of the 2011 official record of the city administration, it comprises 546,219 residents. In the sub city, there are 27 preparatory and secondary schools of these 52% are private, 41% governmental, and 7% missionary schools. Among the governmental schools, only five are preparatory level. In these schools, there are a total of 26,177 students (70% in government schools and 30% in private schools). In total of 19 preparatory schools (5 public and 14 private), public schools have 4305 preparatory level students and private schools have 2084 students. In densely populated squatter settlement areas of the sub-city, the arrival of rural-urban migrants and the encampment of daily laborers increased the prevalence of different substances and production of local drugs sold in roadsides. Khat-selling shops and drug-dealing centers also flourished around schools in the sub city.

### Sample size determination and sampling techniques

The sample size was determined using single population proportion formula considering these assumptions: prevalence of substance use in North Wollo, Woldia preparatory school (34.6%) [15], 95% CI, 5% level of significance, and 5% marginal error.

$$n=\frac{{\left({\mathrm{Z}}_{\upalpha\left/2\right.}\right)}^2\ \mathrm{P}\left(1-\mathrm{P}\right)}{{\mathrm{d}}^2}=\frac{(1.96)^2\ 0.346\left(1-0.346\right)}{(0.05)^2}=348$$

Considering a 10% non-response rate, the final sample size became 383. The study subjects were selected using simple random sampling technique.

### Data collection tools and procedures

Data were collected using a pre-tested substance use questionnaire adopted from the UN toolkit module 3 on conducting school surveys on drug abuse [16]. The questionnaire specifically constituted socio-demographic variables, participants’ substance use, and associated factors such as family, community, school, and environmental factors that influence substance use of students. The researcher provided 1-day training for four facilitators who were college students and guided and closely followed-up with them during the data collection process. The role of facilitators includes time arrangement to reach-out preparatory students, informing the students about the purpose of the study, taking consent from the students, providing explanation for some questions raised by the students during filling of the questionnaire, collecting the filled questionnaire from the students, and check completeness of each questionnaire. The data collection tool was prepared in English and then translated into Amharic to collect data. To differentiate data collected from public and private schools, a special code was used. The researcher supervised the overall data collection process and checked the completeness of the data in the field.

### Description of study variables

The primary outcome variable of this study was substance use. In this study, substance use was defined as taking one or more of the commonly used substances in Ethiopia, such as alcohol (including local liquors like tej, tella, areki), cigarette, khat, shisha, cannabis, and other for different reasons. Students who took one or more of the aforementioned commonly used substances within 30 days prior to the data collection period were taken as current substance users, whereas students who used substances at least once in their lifetime were considered as ever or lifetime-users. The independent variables considered in this study were respondents’ socio-demographic factors ( such as age, sex, religion, parental education, and income level), individual factors (such as perception toward substance use, religiosity, friends’ substance use, peer connectedness, and having excess pocket money), family-related factors (such as family substance use history, parental norm on substance use, family attachment level, and family living arrangement), school-related factors (such as academic performance, school substance use regulation, and school substance controlling system), and environmental factors (such as availability of substances in residential areas, community norms on substance use, and availability of substance selling shops).

### Data management and statistical analysis

Data were entered using EPI-INFO version 3.3 statistical package and export to Statistical Package for Social Sciences (SPSS) version 20 for further analysis. Data were presented by frequencies and percentages for most variables. Univariable binary logistic regression analysis was used to choose variables for the multivariable binary logistic regression analysis on the basis of *P* value less than 0.2. Multivariable binary logistic regression analysis was fitted to control the possible effect of confounders to identify statistically significant variables on the basis of AOR with 95% CI and *P* value less than 0.05.

## Results

### Socio-demographic information

The questionnaires were distributed for a total of 383 students in four randomly selected schools; among them 374 carefully and correctly filled and returned it to the facilitators with a response rate of 97.7%. Two hundred forty-two (64.7%) of the students were aged between 15 and 19 years. The mean age of the participants was 18.1 years (SD ± 1.077 years). From the total 374 respondents, 194 (51.9%) were female. Two hundred eleven (56.4%) of them were grade 11. Two hundred ninety-nine (79.9%) of the study participants were from public schools (Table 1).

**Table 1:** Socio-demographic characteristics of study subjects in Kolfe-Keraniyo sub-city, Addis Ababa, July 2020 (*n* = 374)

Variables	Frequency	Percent
Age		
15-19	242	64.7
20-24	132	35.3
Sex		
Male	180	48.1
Female	194	51.9
Grade		
11	211	56.4
12	163	43.6
Ethnicity		
Amhara	103	27.5
Oromo	106	28.3
Tigre	43	11.5
Guraghe	71	19.0
Others	51	13.6
Religion		
Orthodox	174	46.5
Muslim	119	31.8
Protestant	74	19.8
Others	7	1.9
School type		
Public	299	79.9
Private	75	20.1
Educational status of mother		
Illiterate	33	8.8
Primary	109	29.1
Secondary	91	24.3
Diploma	79	21.1
Degree and above	62	16.6
Educational status of mother		
Illiterate	92	24.6
Primary	149	39.8
Secondary	71	19.0
Diploma	55	14.7
Family monthly income (in Birr)		
≤ 5000	116	31.0
5001-7000	83	22.2
7001-10000	82	21.9
> 10000	40	10.7
Occupation of mother		
House wife	277	74.1
Merchant	33	8.8
Government employee	39	10.4
Private employee	24	6.4
Pensioner	1	0.3
Occupation of father		
Merchant	85	22.7
Government employee	110	29.4
Private employee	116	31.0
Pensioner	59	15.8
Unemployed	4	1.1
Condition of family		
Living together	275	73.5
Divorced	53	14.2
One/both died	40	10.7
No answer	6	1.6
If not with both parents with whom presently live		
With father only	51	13.6
With mother only	31	8.3
With relatives	29	7.8
With friends	10	2.7
Connection with friends		
Strong	205	54.8
Normal	164	43.9
Weak	5	1.3
Does your parents know everything about you		
Yes	242	64.7
No	132	35.3
Connection with parents		
Strong	211	56.4
Normal	136	36.4
Weak	27	7.2
Do you visit religious centers		
Yes	341	91.2
No	33	8.8
Frequency of visiting religious centers		
Visit daily	56	15.0
Twice a week	71	19.0
Once a week	81	21.7
As needed	135	36.1

### Prevalence of substance use

**Fig. 2:**
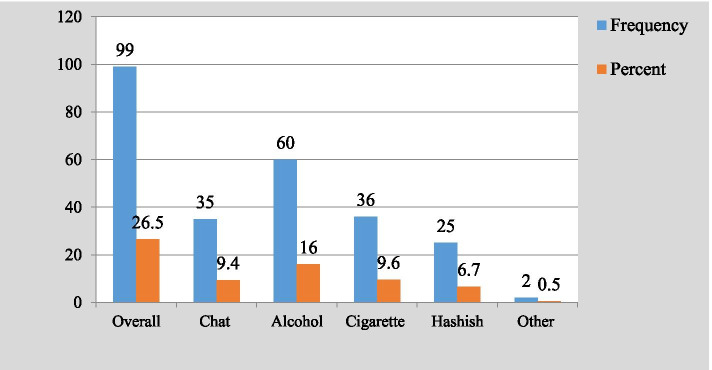
Prevalence of lifetime substance use among preparatory students in Kolfe-Keraniyo sub-city, Addis Ababa, July 2020 (*n* = 374)

**Fig. 3:**
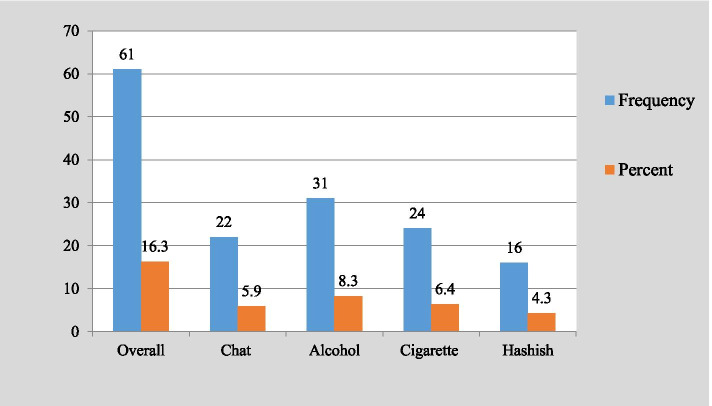
Prevalence of current substance use among preparatory students in Kolfe-Keraniyo sub-city, Addis Ababa, July 2020 (*n* = 374)

From a total of 374 students, 99 students reported that they ever used at least one substance. The overall prevalence of lifetime substance use was, therefore, found to be 26.5% (95% CI, 22.2, 30.7%). Participants often used alcohol [60 (16.0%)], cigarette [36 (9.6%)], khat [35 (9.4)], and hashish [25 (6.7%)] (Fig. 2). Two students reported that they used all the aforementioned substances. Six students reported that they used alcohol, cigarette, and hashish. Forty (10.7%) of the students reported that they used two of the aforementioned substances. Regarding current substance use, 16.3% (95% CI, 12.8, 19.8) of the respondents reported that they currently using the substance, of these 22 (5.9%) chew khat, 31 (8.3%) drunk alcohol, 24 (6.4%) smoked cigarette, and 16 (4.3%) took hashish (Fig. 3).

### Frequency of substance use and associated reasons

Nine (2.4%) of the respondents used substances more than twice a day. About 78 (20.9%) of the participants reported that they used substances with their friends and 81 (21.7%) of them were influenced by their friends to start using a substance. Similarly, 42 (11.2%) of them reported that they use a substance in their friends’ houses (Table 2).

**Table 2:** Frequency of for substance use among preparatory students in Kolfe-Keraniyo sub-city, Addis Ababa, July 2020 (*n* = 374)

Variables	Frequency	Percent
Frequency of substance use		
Twice and above a day	9	2.4
Once a day	21	5.6
Twice and above a week	8	2.1
Weekly	23	6.1
As needed	38	10.2
With whom substance used		
Alone	8	2.1
Friends	78	20.9
Family members	13	3.5
Who influence to start substance		
Family members	16	4.3
Friends	81	21.7
Others	2	0.5
Place of substance use		
Home	13	3.5
School	7	1.9
Shopping centers	37	9.9
In friends’ house	42	11.2

### Reasons for substance use

#### Individual reasons for substance use

**Fig. 4:**
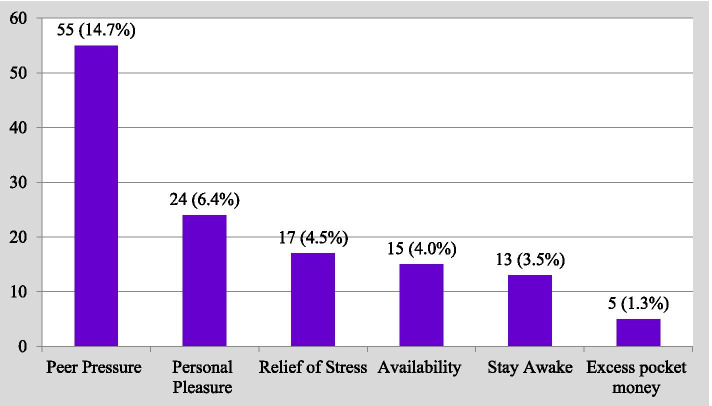
Reasons for substance use among preparatory students in Kolfe-Keraniyo sub-city, Addis Ababa, July 2020 (*n* = 374)

Availability of substance retailing shops in the residential and school areas [84 (22.5%)], peer pressure [55 (14.7%)], for personal pleasure [24 (6.4%)], desire to get relief of stress [17 (4.5%)], to stay awake [13 (3.5%)], and having excess pocket money [5 (1.3%)] were the reported reasons to use substances. Three hundred thirty-three (89.0%) of respondents confirmed that substance use is harmful (Fig. 4).

#### Family-related reasons for substance use

Participants were requested about the stand of their parents regarding their substance use and whether anyone of their family members using the substance. Accordingly, 360 (96.2%) of the participants reported that their parents did not allow them to use a substance. Regarding the substance use history of their family members, 129 (34.5%) reported that their family members use a substance. The study revealed that alcohol [71 (19.0%)], khat [64 (17.1%)], and cigarette [53 (14.2%)] were the most common substances by parents of respondents’ use is harmful (Table 3).

**Table 3:** Family-related reasons for substance use of preparatory students in Kolfe-Keraniyo sub-city, Addis Ababa, July 2020 (*n* = 374)

Variables	Frequency	Percent
Substance use stand of parents		
Disapproval	360	96.2
Neutral	3	0.8
Family member using substance		
Yes	129	34.5
No	245	65.5
Parents use substance		
Yes	18	4.8
No	356	95.2
Father using substance		
Yes	93	24.9
No	281	75.1
Mother using substance		
Yes	19	5.1
No	355	94.9
Sibling using substance		
Yes	45	12.0
No	329	88.0
Family member chew khat		
Yes	64	17.1
No	310	82.9
Family member drink alcohol		
Yes	71	19.0
No	303	81.0
Family member smoke cigarette		
Yes	53	14.2
No	321	85.8

#### Substance use and school-related reasons

About 50 (13.4%) of the study participants assumed that substance use improves academic performance. Two hundred thirty-nine (63.9%) of the participants reported that their school has rules to control substance use and accordingly 40 (10.7%) study participants noticed that students were completely dismissed due to substance use in school. Three hundred eleven (83.2%) of the respondents reported that the substance use controlling system in their school was in effect. Two hundred forty-nine (66.6%) of the study participants reported that their homeroom teacher consistently supervised them. Regarding their extracurricular engagement at school, 81 (21.7%) of the participants were a member of school mini-media and 198 (52.9%) of the respondents reported that school mini-medias sensitized students on the effect of substance use (Table 4).

**Table 4:** School-related reasons for substance use of preparatory students in in Kolfe-Keraniyo sub-city, Addis Ababa, July 2020 (*n* = 374)

Variables	Frequency	Percent
Improve academic performance		
Yes	50	13.4
No	324	86.6
School has substance use controlling rule		
Yes	239	63.9
No	135	36.1
Measures taken by school on substance use		
Advice give	102	27.3
Warning	127	34.0
Temporary dismissal	105	28.1
Complete dismissal	40	10.7
In effective school substance use controlling rule		
Yes	311	83.2
No	63	16.8
Homeroom teacher supervise		
Yes	249	66.6
No	125	33.4
Being member of school mini-media		
Yes	81	21.7
No	293	78.3
Awareness given by mini-media on		
Yes	198	52.9
No	176	47.1

#### Environmental reasons for substance use

Out of 374 respondents, 229 (61.2%) reported that availability of substance retailing shops around school made them use a substance. One hundred thirty-nine (37.2%) of respondents reported that the shops sell substances openly to students. One hundred ninety-one (51.1%) of the respondents reported that substance retailing shops are available in their residential area. Three hundred twelve (83.4%) of the respondents reported that their community has a disfavoring stand toward substance use (Table 5).

**Table 5:** Environment-related reasons for substance use among preparatory students in Kolfe-Keraniyo sub-city, Addis Ababa, July 2020 (*n* = 374)

Variables	Frequency	Percent
Substance selling shops availed around school		
Yes	229	61.2
No	145	38.8
Shops sell substance openly to students		
Yes	139	37.2
No	235	62.8
Availability of substance in residential area		
Yes	191	51.1
No	183	48.9
Community stand on substance use		
Disfavoring	312	83.4
Neutral	10	2.7

#### Protective reasons from substance use

**Fig. 5:**
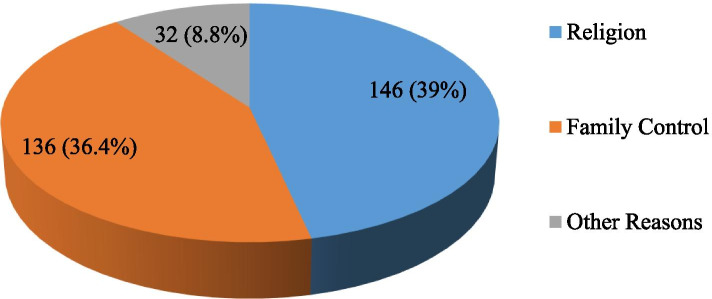
Reasons prevent students from substance use in Kolfe-Keraniyo sub-city, Addis Ababa, July 2020 (*n* = 374)

Research participants were asked about protecting reasons from substance use. Accordingly, 146 (39.0%) of respondents reported that their religion made them not to use a substance. Similarly, 136 (36.4%) of respondents were found not using substances due to family control. Thirty-two (8.8%) of the respondents also mentioned other factors (like, because they did not want and believe substance use is harmful) which influenced them from using substances (Fig. 5).

#### Factors associated with substance use

This study revealed that gender, age, grade level, having a family member who chews khat, drinks alcohol, and smoke cigarette, knowing of parents of whereabouts of their children, knowing of parents everything about their children associated with substance use. Besides, believing that substance use improves academic performance, having substance use controlling rule of school, measures taken by schools for using substances, ineffective (poor) school substance use controlling rule, supervision of homeroom teacher, being a member of school mini-media, and awareness of mini-media on substance use were also associated with substance use (Table 6).

**Table 6:** Factors associated with substance use among preparatory school students in Kolfe-Keraniyo sub-city, Addis Ababa, July 2020 (*n* = 374)

Explanatory variables	Substance use		COR (95% CI)	AOR (95% CI)
	Yes	No		
Age				
16-18	56	186	0.623 (0.389, 0.998)	
Above 19	43	89	1	
Sex				
Male	64	116	2.51 (1.556, 4.036)	3.326 (1.284, 8.613)*
Female	35	159	1	
Grade level				
11	43	168	0.49 (0.307, 0.779)	
12	56	107	1	
Family member chew khat				
Yes	28	36	2.62 (1.495, 4.585)	2.866 (1.161, 7.070)*
No	71	239	1	
Family member smoke cigarette				
Yes	28	25	3.94 (2.164, 7.188)	
No	71	250	1	
Family member drink alcohol				
Yes	46	25	8.68 (4.908, 15.349)	3.958 (1.704, 9.196)**
No	53	250	1	
Parents know your whereabouts				
Yes	32	47	0.049 (0.028, 0.088)	
No	69	28	1	
Parents know everything about you				
Yes	7	235	0.013 (0.006, 0.030)	0.005 (0.001, 0.026)***
No	92	40	1	
Substance use improves academic performance				
Yes	35	15	9.479 (4.881, 18.410)	
No	64	260	1	
Poor school substance use controlling rule				
Yes	89	222	2.125 (1.035, 4.361)	6.644 (1.863, 23.687)**
No	10	53	1	
Member of school mini-media				
Yes	10	71	0.323 (0.159, 0.655)	0.177 (0.048, 0.657)*
No	89	204	1	
Mini-media aware on substance				
Yes	39	60	0.645 (0.410, 1.045)	
No	138	137	1	
Shops sell substance openly				
Yes	69	70	6.736 (4.056, 11.187)	
No	30	205	1	
Substance available at residential areas				
Yes	81	110	6.750 (3.837, 11.874)	2.934 (1.303, 6.606)**
No	18	165	1	

*Statistically significant at *P* < 0.05

**Statistically significant at *P* < 0.01

***Statistically significant at *P* < 0.001 Hosmer and Lemeshow test = 0.473

The current study depicted that substance use was statistically associated with sex of students. The odds of substance use were 3.3 times more likely to be higher among male students compared with female (AOR, 3.326; 95% CI, 1.284, 8.613). Substance use among students was also significantly associated with history of substance use in their family member. Students whose families have history of khat chewing are 2.9 times more likely exposed to substances than those whose family members do not chew khat (AOR, 2.866; 95% CI, 1.161, 7.070). Similarly, respondents whose family members drink alcohol were 4.0 times more likely to use substance than respondents whose family members do not have alcohol use history (AOR, 3.958; 95% CI, 1.704, 9.196). Similarly, availability of substance in the residential area increased likelihood of substance use (AOR, 2.934; 95% CI, 1.303, 6.606). This study explored that child-family relationship was associated with the likelihood of children’s substance use. Accordingly, students of those parents who know everything about them were 99.5% less likely to use substance than those whose parents do not know everything about them (AOR, 0.005, 95% CI, 0.001, 0,026). The study also explored that school environment was associated with substance use. The probability of substance use among students was 6.6 times more likely to be higher in schools with poor substance use controlling rule (AOR, 6.644; 95% CI, 1.863, 23.687). The study also reported that participation in school mini-medias was statistically associated with substance use. Students who participated in school mini media clubs were 82.3% less likely to use substance compared with their counterparts (AOR, 0.177; 95% CI, 0.048, 0.657).

## Discussion

This study depicted that the prevalence of lifetime and current substance use among students of preparatory schools in Kolfe-Keraniyo sub-city, Addis Ababa, was 26.5% (95% CI, 22.2, 30.7%) and 16.3% (95% CI, 12.8, 19.8) respectively. Among the lifetime users, 60 (16.0%) drunk alcohol, 36 (9.6%) smoked cigarettes, 35 (9.4%) chew khat and 25 (6.7%) took hashish. While among current substance users, 31 (8.3%) drunk alcohol, 24 (6.4%) smoked cigarettes, 22 (5.9%) chew khat, and 16 (4.3%) took hashish. The overall prevalence of substance use in this study was closely similar to the study findings of researches conducted in Wolayta (28.6%) [17] and Sudan (31%) [18].

The prevalence of ever smoking cigarettes was similar to the study conducted in urban and rural secondary schools in Ethiopia (9%) [19]. Furthermore, the current prevalence of cigarette smoking in this study was in line with a report from Wereta town high school students (6.8%) [20]. Regarding the prevalence of lifetime hashish use, the report of this study was similar to the study conducted in secondary schools of Addis Ababa, Ayertena, Ethiopia (5.9%) [21] and Southern Iran (5.2%) [10], Sudan (4.9%) [18], and Nigeria (3%) [1]. The current khat chewing prevalence was close to the report obtained from Addis Ababa (4.2%) [21].

The prevalence of lifetime and current substance use reported in the current study was lower than findings of studies in different parts of Ethiopia, such as Addis Ababa (24.6% current prevalence) [21], North Wollo (34.6%) [15], Bale (34.8%) [22], Woreta town (65.4%) lifetime prevalence and (47.9%) current prevalence [23], and Nigeria was 66% [1]. The discrepancy observed might be due to geographic and cultural differentials in areas of study and sample size differences. The prevalence of ever and current use of alcohol in this study was lower than a study conducted in urban and rural students in Ethiopia (28.6% and 14.1%) [19] respectively. Furthermore, the current use of alcohol in this study was lower than North Wollo, Woldia preparatory school students (23.5%) [15] and Bale preparatory students (23.6%) [22]. Such a sizeable difference in substance use might be due to the cultural difference in the acceptability of drinking alcohol in study areas. Furthermore, recent enforcement restriction of televised and media advertisement of alcohol might contribute for decline in prevalence of alcohol consumption of students.

The prevalence of ever cigarette smoking in this study was lower than the prevalence reported by a study in Woreta high school students (23%) [23] and high school students in Iraq (27.6%) [14] and higher than Nigeria high school students (3%) [1]. The reason for differences in prevalence might be sample population and cultural differences. This study focused only on preparatory students (grades 11 to 12) while the remaining two covered the whole high school students. The current cigarette smoking prevalence in this study was higher than reports from urban and rural Ethiopia (2.6%) [21], Bale (2.6%) [22], Woldia (3.3%) [15], and Addis Ababa (0.8%) [21]. The variations observed in this regard might have resulted from cultural and geographic differences.

The prevalence of ever and current khat chewing reported in this study was lower than reports of studies in Ataye secondary school students (15.4% and 13.3%) respectively [24], in Bale (17.1%) [22], in Woldia (23.5%) [15], and in Woreta (13.8%) [23]. This difference might be due to the fact that chewing khat in other parts of Ethiopia has been embedded in the culture and social acceptability and also accessibility of khat in farm lands also aggravate khat chewing among students. While in Kolfe-Keraniyo, this study was conducted on, students can only access it if they afford the price.

This research identified that substance use is associated with the sex of students. Male students had more odds of substance use than females. This report was similar to the study done in different parts of Ethiopia, such as Bale [22], Ambo [19], Woreta [22], and Ataye [24]. This might be due to socially constructed gender role divisions; relative freedom males have to go out of home increased substance use of male students and tight family control and social stigma toward substance use reduced likelihood of substance use among female students. Besides, a male takes greater risk than a female due to gendered social norms, nature, and physiology [23].

In this study, family history of substance use was associated with substance use among students. Parents who use a substance in front of their children became role models to their children and made children believe that substance use of adults is acceptable behavior [23]. Similarly, having a family member who drinks alcohol and chew khat is strongly associated with substance use of students [22]. This study also revealed that the poor school substance use controlling rule is associated with substance use among students. In situations when students are left unchecked, they bring substance into school in their bag, and exchange and use it in all possible areas including toilets [13]. This finding was in line with a study in Ambo secondary school students [25].

This study reported that the availability of substances in residential areas was significantly associated with increased substance use. This finding was similar to previous studies conducted in Ambo town [25] and Debre Markos, Ethiopia [26]. Similarly, students with an intense parent-child relationship were less likely to use the substance. This finding was comparable with the study conducted in Nigeria [1]. Parents who were supportive and spent more time with their children increased openness of children to share everything to their parents and parents therefore able to monitor and control unwanted behaviors of their children including substance use [13].

In this study, the pro-social involvement of students, particularly membership to school mini-media protected students from a substance. This finding is supported by a study in Woreta, Ethiopia [23]. This implies that as students became prone to pro-social and extracurricular activities both for their benefit and the benefit of the larger public; they are less likely to use substances. As students spent their free time participating in extracurricular activities in schools and the community, they enter into a self-coaching and mutual monitoring framework reducing the likelihood of substance use [27, 28].

As a limitation, this study might be affected by social desirability bias. Students might not report substance use because substance use in Ethiopian culture is not acceptable. We used self-administered interview questionnaire to minimize this bias. Moreover, privacy was assured during data collection time.

## Conclusion

This study found that a quarter of the study participants were substance users. Alcohol, khat, and cigarette were widely used substances. Gender, parent-child relationship, substance use history of family members, school substance use controlling rules, school mini-media and pro-social involvement, and availability of substance retailing shops were strongly associated with substance use. Strengthening school rules on substance use and school-based programs to adolescent drug abuse prevention should be in place as school-based efforts are efficient in that they offer access to large numbers of students. The local health office should enforce the retailing shops not to sell substances to the students and if possible substance retailing shops near school should be closed. The local government has to plan social interventions geared by creating recreations alternatives and opportunities for youth. Social resistance skills to increasing adolescent’s awareness of the various social influences that support substance use and teaching them specific skills for effectively resisting both peer and media pressures to smoke, drink, or use drugs is also important.

## Data Availability

Data will be made available upon requesting the primary author.

## Abbreviations

AOR: Adjusted odds ratio
CI: Confidence interval
COR: Crude odds ratio
COPD: Chronic obstructive pulmonary diseases
CVD: Cardio vascular disease
HIV: Human immune deficiency virus
SPSS: Statistical Program for Social Science
UNODC: United Nations Office on Drugs and Crime
WHO: World Health Organization

## References

[1] Ogunsola OO, Fatusi AO. Risk and protective factors for adolescent substance use: a comparative study of secondary school students in rural and urban areas of Osun State, Nigeria. International journal of adolescent medicine and health. 2016; 29(3). 10.1515/ijamh-2015-0096 26824975

[2] W.H.O. Global status report on alcohol and health. Geneva. 2014;Geneva: (https://www.who.int/iris/bitstre am/10665/112736/1/9789240692763_eng.pdf, Accessed 12 February 2020.

[3] American Lung Association. Facts about Hookahexternal. Washington: American Lung Association, 2007. Available at https://www.lung.org/quit-smoking/smoking-facts/health-effects/facts-about-hookah. Accessed on 30 May 2021.

[4] United States Drug Enforcement Administration (DEA). Drugs of Abuse A DEA RESOURCE GUIDE 2020 Edition . Available at https://www.dea.gov/sites/default/files/2020-04/Drugs%20of%20Abuse%202020-Web%20Version-508%20compliant-4-24-20_0.pdf. Accessed on 31 May 2021.

[5] Gebremariam TB, Miruts KB, Neway TK (2018). Substance use and associated factors among DebreBerhan university students, central Ethiopia, substance abuse, treatment, prevention and policy. Substance Abuse Treat Prev Policy.

[6] Samuel S, Berhanu A (1991). The microbiology of tella fermentation. Sinet..

[7] Steinkraus K (2018). Handbook of indigenous fermented foods, revised and expanded.

[8] Wedajo Lemi B. Microbiology of Ethiopian Traditionally Fermented Beverages and Condiments. International journal of microbiology. 2020:1478536. 10.1155/2020/1478536. 10.1155/2020/1478536 PMC7042527 32148508

[9] Alemu F, Amhaselassie T, Kelbessa U, Elias S (1991). Methanol, fusel oil, and ethanol contents of some Ethiopian traditional alcoholic beverages. Sinet..

[10] Heydari S, Izedi S, Sarikhani Y, Kalani N, Akbary A, Miri A (2015). the prevalence of substance use and associated risk factors among university students in the city of Jahrom, Southern Iran. Int J Higher Risk Behav Addict.

[11] Igwe W, Ojinnaka N, Ejiofor S, Emechebe G, Ibe B (2009). Socio-demographic correlates of psychoactive substance abuse among secondary school students in Enugu, Nigeria. Eur J Soc Sci.

[12] Abasiubong F, Atting I, Bassey E, Ekott J (2008). A comparative study of use of psychoactive substances amongst secondary school students in two local government areas of Akwa Ibom state, Nigeria. Nigerian J Clin Pract.

[13] Layla A, Naseeba AO, Hisham E, Ahmed E-K, Shamil W, Amna A (2015). Adolescents’ perception of substance use and factors influencing its use: a qualitative study in Abu Dhabi. J R Soc Med Open.

[14] Mahmood N, Othman S, Al-Tawil N, Al-Hadithi T (2019). Substance use among high school students in Erbil City, Iraq: prevalence and potential contributing factors. East Mediterr Health J.

[15] Gobeje A, Measo G, Ajeb A, Chanie T (2019). Prevalence of substance use and associated factors among preparatory students of N/Wollo Woldia Town, North East Ethiopia, 2015. Acta Scientific Nutr Health.

[16] UNITED NATIONS OFFICE ON DRUGS AND CRIME. Conducting school surveys on drug abuse. Global Assessment Programme on Drug Abuse (GAP) Toolkit Module 3, 2003. Available at https://www.unodc.org/documents/GAP/GAP%20Toolkit%20Module%203%20ENGLISH.pdf. Accessed on 15 February 2020.

[17] Mekonen T, Fekadu W, Mekonnen T (2017). Substance use as a strong predictor of poor academic achievement among university students. Hindawi Psychiatry.

[18] Osman T, Victor C, Abdulmoneim A, Mohammed H, Abdalla F, Ahmed A, et al. Epidemiology of substance use among university students in Sudan. J Addict. 2016:2476164. 10.1155/2016/2476164. 10.1155/2016/2476164 PMC4783543 27006856

[19] Getachew S, Lewis S, Britton J, Deressa W, Fogarty AW. Prevalence and risk factors for initiating tobacco and alcohol consumption in adolescents living in urban and rural Ethiopia. Public health. 2019;174:118-126. 10.1016/j.puhe.2019.05.029 PMC7684154 31330474

[20] Birhanu AM, Bisetegn TA, Woldeyohannes SM (2014). High prevalence of substance use and associated factors among high school adolescents in Woreta Town, Northwest Ethiopia: multi-domain factor analysis. BMC Public Health.

[21] Henok A. Exploring the trends and challenges of substance abuse among Ayer Tena Secondary School students in Addis Ababa; 2015.AAU Institutional Repository. https://www.localhost:80/xmlui/handle/123456789/1772.

[22] Dida N, Kassa Y, Sirak T, Zerga E, Dessalegn T. Substance use and associated factors among preparatory school students in Bale Zone, Oromia Regional State, Southeast Ethiopia. Harm reduction journal. 2014;11:21. 10.1186/1477-7517-11-21 PMC4131802 25108629

[23] Birhanu AM, Bisetegn TA, Woldeyohannes SM. High prevalence of substance use and associated factors among high school adolescents in Woreta Town, Northwest Ethiopia: multi-domain factor analysis. BMC Public Health. 2014; 14:1186. 10.1186/1471-2458-14-1186 PMC4289242 25410657

[24] Lakew A, Tariku B, Deyessa N, Reta Y (2014). Prevalence of Catha edulis (Khat) Chewing and Its Associated Factors among Ataye Secondary School Students in Northern Shoa. Ethiopia. Advances in Applied Sociology..

[25] Mekuria M, Girma T, Birhanu A. Assessment of Substance Abuse and Associated Factors among Secondary School Students in Ambo Town, Ethiopia, 2018. J Addict Res Ther. 2019; 10:383. 10.4172/2155-6105.1000385.

[26] Tesfahun A, Gebeyaw T, Girmay T. Assessment of substance abuse and associated factors among Students of Debre Markos Poly Technique College in Debre Markos Town, East Gojjam Zone, Amhara Regional State, Ethiopia. Global Journal of Medical research Pharma, Drug Discovery, Toxicology and Medicine. 2013;13(4): 4-15.

[27] Ostaszewski K. Inadequate Models of Adolescent Substance Use Prevention: Looking for Options to Promote Pro-Social Change and Engagement. Substance use & misuse. 2015, 50(8-9):1097-1102. 10.3109/10826084.2015.1010897 26222691

[28] Cleveland MJ, Feinberg ME, Bontempo DE, Greenberg MT: The role of risk and protective factors in substance use across adolescence. The Journal of adolescent health. 2008; 43(2):157-164. 10.1016/j.jadohealth.2008.01.015 PMC2518980 18639789

